# Early mNGS testing for diagnose and prognostic prediction of early onset pneumonia among in-hospital cardiac arrest patients undergoing extracorporeal cardiopulmonary resuscitation

**DOI:** 10.3389/fcimb.2024.1382273

**Published:** 2024-11-08

**Authors:** Rui-ming Guo, Xing-xing Li, Yi-heng Zhou, Yi-juan Liu, Jun Li, Guo-wei Fu, Hui Zhao, Xin Zhang, Yang-chao Zhao

**Affiliations:** ^1^ Department of Cardiac Surgery, The First Affiliated Hospital of Zhengzhou University, Zhengzhou, Henan, China; ^2^ Department of Extracorporeal Life Support Center, Department of Cardiac Surgery, The First Affiliated Hospital of Zhengzhou University, Zhengzhou, Henan, China; ^3^ Henan Medical School of Zhengzhou University, Zhengzhou, Henan, China; ^4^ Department of Biobank, The First Affiliated Hospital of Zhengzhou University, Zhengzhou, Henan, China

**Keywords:** in-hospital cardiac arrest, extracorporeal cardiopulmonary resuscitation, metagenomic next-generation sequencing, early onset pneumonia, prognosis

## Abstract

**Objectives:**

Metagenomic next-generation sequencing (mNGS) is emerging as a novel diagnostic technology for various infectious diseases; however, limited studies have investigated its application in etiological diagnosis of early onset pneumonia (EOP) among patients undergoing extracorporeal cardiopulmonary resuscitation (ECPR) following in-hospital cardiac arrest (IHCA), The clinical significance of early mNGS in predicting short-term prognosis of IHCA patients after ECPR remains unclear.

**Methods:**

This retrospective study included 76 patients with IHCA who underwent ECPR at the First Affiliated Hospital of Zhengzhou University from January 2018 to December 2022. Baseline characteristics and etiological data of all patients during their hospitalization were collected and statistically analyzed. The primary outcome of this study was the diagnosis of EOP, while the secondary outcomes included successful extracorporeal membrane oxygenation (ECMO) weaning and survival at discharge. Additionally, the characteristics of bronchoalveolar lavage fluid (BALF) flora in these patients were compared by analyzing both mNGS results and culture results.

**Results:**

Multivariate logistic regression were employed to analyze the predictors of ECMO weaning failure, mortality at discharge, and the incidence of EOP. Ultimately, patients with lower SOFA scores on admission [OR (95%CI): 1.447 (1.107-1.890), p=0.007] and those who underwent early mNGS testing within 48 hours after ECPR [OR (95%CI): 0.273 (0.086-0.865), p=0.027] demonstrated a higher probability of successful weaning from ECMO. Patients with higher SOFA scores on admission [OR (95%CI): 2.404 (1.422-4.064), p=0.001], and elevated lactate levels [OR (95%CI): 1.176 (1.017-1.361), p=0.029] exhibited an increased likelihood of mortality at discharge. Furthermore, early mNGS detection [OR (95%CI): 0.186 (0.035-0.979), p=0.047], and lower CRP levels (48h-7d after ECMO) [OR (95%CI):1.011 (1.003-1.019), p=0.006] were associated with a reduced incidence of EOP. In addition, the pathogens detected by mNGS within 48 hours after ECPR were mainly oral colonizing bacteria and viruses, and viruses were in the majority, while all BALF cultures were negative. In contrast, between 48 hours and 7 days after ECPR, BALF cultures were positive in all EOP patients.

**Conclusions:**

Early mNGS testing to identify microbial flora facilitates timely adjustment of antibiotic regimens, thereby reducing the incidence of EOP and improving short-term prognosis in patients undergoing ECPR following IHCA.

## 1 Introduction

In-hospital cardiac arrest (IHCA) is a prevalent and rapidly progressive condition with a high mortality rate, reaching up to 70%. Despite its fatalness, IHCA has received limited attention compared to other high-risk cardiovascular diseases like myocardial infarction and out-of-hospital cardiac arrest (Richardson et al., 2021; Andersen et al., 2019; Schluep et al., 2018). Extracorporeal cardiopulmonary resuscitation (ECPR), which involves the use of extracorporeal membrane oxygenation (ECMO), offers hemodynamic support to maintain vital organ perfusion in the presence of persistent cardiovascular impairment, potentially improving survival after IHCA (Richardson et al., 2021; Vakil et al., 2021). Patients with IHCA often experience severe hypoxemia or face constant risks of aspiration due to their inability to clear upper airway secretions and gastric reflux independently. Rapid tracheal intubation becomes essential for minimizing low flow duration during ECPR implementation (Ryu et al., 2015). However, achieving both prompt intubation and impeccable asepsis can be challenging when urgently performing ECPR on IHCA patients. Furthermore, these patients frequently require continuous ventilator-assisted ventilation, indwelling veno-arterial ECMO (VA ECMO) catheters, and sometimes continuous renal replacement therapy (CRRT) tubes and intra-aortic balloon pump (IABP) tubes-all contributing as risk factors for infection development (Raad et al., 2007). A proportion of patients die from infectious shock secondary to IHCA.

In addition, ECMO is associated with the up-regulation of inflammatory mediators, such as cytokines, endotoxins, and oxygen-derived free radicals, and even contributes to the development of systemic inflammatory response syndrome (SIRS) (François et al., 2019; Millar et al., 2016; Mongardon et al., 2011; Perbet et al., 2011; Shiba et al., 2020). Recent studies have demonstrated that levels of C-reactive protein (CRP), a crucial inflammatory factor, are correlated with early-onset pneumonia (EOP) in ECPR patients (Shiba et al., 2020). In the early stage of EOP, patients may present with elevated levels of white blood cells, CRP, fever, etc. However, prompt administration of antibiotics can potentially obscure the true pathogen and mask the positive rate of traditional pathogen tests such as blood culture. Consequently, achieving an accurate etiological diagnosis becomes a challenging task (François et al., 2019). In patients undergoing ECPR for IHCA, there is a significantly increased risk of infection necessitating prompt identification of the pathogen and initiation of antibiotic treatment.

In recent years, metagenomic next-generation sequencing (mNGS) has garnered significant attention in the field of infectious diseases due to its high throughput capacity and rapid turnaround time (Mitchell and Simner, 2019; Wu et al., 2020; Xie et al., 2019; Zhou et al., 2019), substantially reducing clinical diagnosis time. Several studies have highlighted that mNGS testing can identify antimicrobial resistance (AMR) genes and assist clinicians in adjusting antibiotic treatment regimens (Deurenberg et al., 2017; Lefterova et al., 2015). Moreover, the diagnostic accuracy of mNGS remains unaffected by antibiotic use (Grumaz et al., 2019). The feasibility of mNGS in bronchoalveolar lavage fluid (BALF), blood, sputum, transbronchial lung biopsy (TBLB), and even lung tissue biopsy for patients with respiratory tract infections has been well-documented in the literature (Chen et al., 2020; Li et al., 2018, Li et al., 2020; Liu et al., 2019; Wu et al., 2020). However, the utilization of mNGS testing in patients undergoing ECPR remains relatively infrequent, with limited research available and a lack of knowledge regarding its impact on the incidence of EOP and mortality rates among ECPR patients.

This study aims to encompass patients undergoing ECPR for IHCA, analyze their baseline characteristics, evaluate the impact of early mNGS testing on the incidence of EOP and short-term prognosis in such patients, investigate the causative organisms involved, and provide guidance for precise antibiotic utilization in clinical practice.

## 2 Materials and methods

### 2.1 Research population

This study retrospectively included IHCA patients who underwent ECPR between January 2018 and December 2022 in the intensive care unit (ICU) of the First Affiliated Hospital of Zhengzhou University. The enrollment criteria for ECPR are in accordance with Extracorporeal Life Support Organization (ELSO) guidelines (Richardson et al., 2021): 1) age <70 years; 2) witnessed cardiac arrest; 3) first CPR (“no flow interval”) <5 minutes or bystander CPR; 4) initial rhythm of ventricular fibrillation (VF)/pulseless ventricular tachycardia (pVT)/absence of pulsatile electrical activity (PEA); 5) ECMO blood flow arrest <60 minutes “low flow interval” (unless other favorable prognostic features are present, such as intermittent recovery from autonomic circulation, preoperative hypothermia, rejuvenation, or stable vital signs during CPR); 6) intermittent return of spontaneous circulation or recurrent ventricular fibrillation; 7) no previously known life-limiting comorbidities (e.g., end-stage heart failure, chronic obstructive pulmonary disease, end-stage renal failure, liver failure, end-stage disease), and consistent with the patient’s goals of care; and finally,8) no known aortic insufficiency (mild aortic dyskinesia should be ruled out).

**Figure 1:**
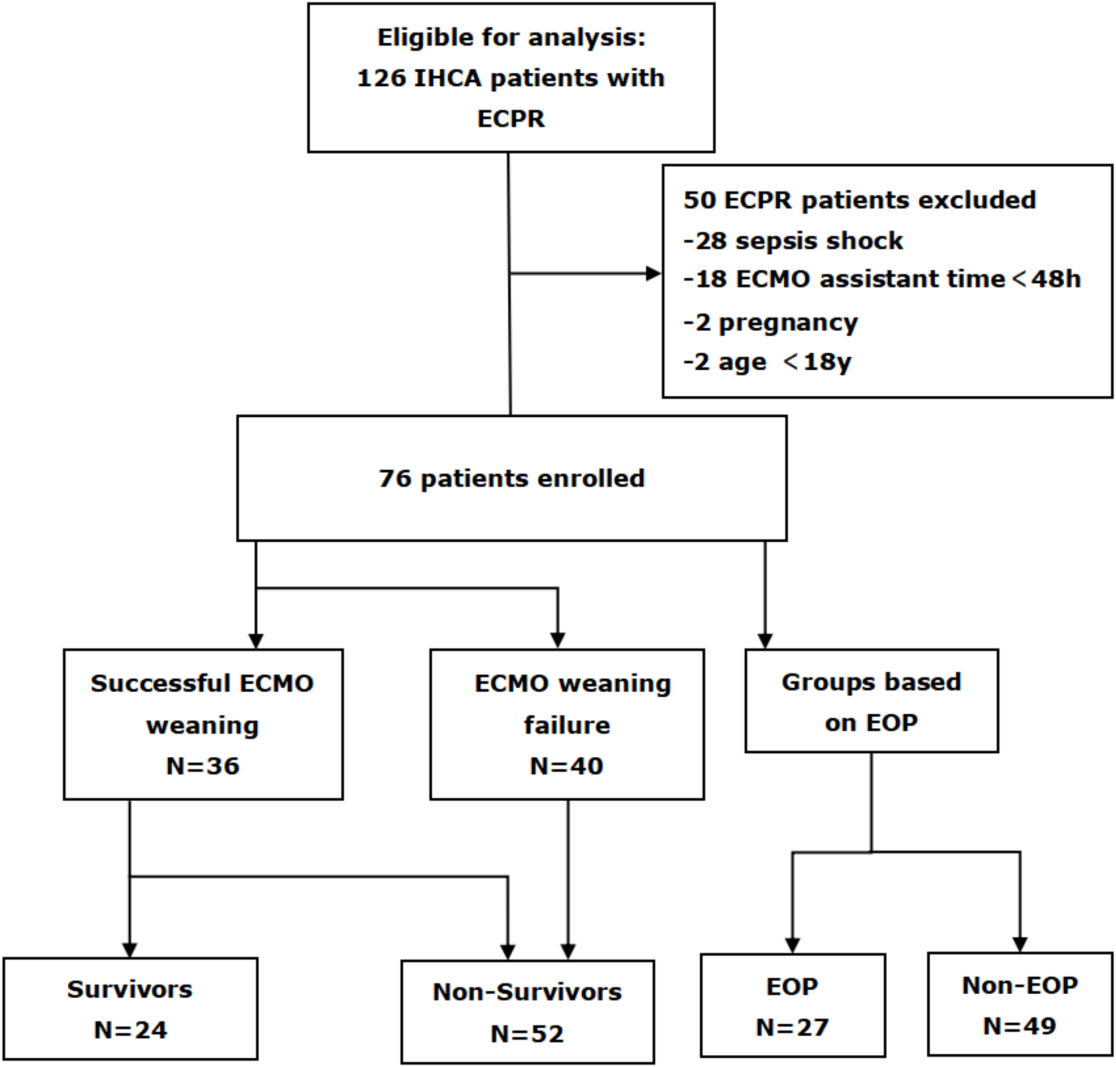
Flow diagram of patient inclusion and exclusion. IHCA, in-hospital cardiac arrest; ECPR, extracorporeal cardiopulmonary resuscitation; ECMO, extracorporeal membrane oxygenation; EOP, early onset pneumonia.

A total of 126 IHCA patients met the criteria for VA ECMO support. Among them, exclusion criteria included 28 patients with cardiac arrest caused by infectious shock, 18 patients who received ECMO assistance for less than 48 hours, as well as two pregnant women and two individuals under the age of 18 (**Figure 1**). After the screening process, a total of 76 eligible patients were ultimately included.

The present study was conducted in compliance with the Declaration of Helsinki and was approved by the Ethics Committee of the First Affiliated Hospital of Zhengzhou University, Zhengzhou, China (no. 2020-KY-429).

### 2.2 Data collection and processing

We collected and documented baseline information and laboratory test data from the electronic medical record system for the enrolled patients. Baseline information encompassed age, gender, body mass index (BMI), smoking history, comorbidities, primary disease, intra-aortic balloon pump (IABP) utilization, sequential organ failure assessment (SOFA) score on admission, time consumption for VA-ECMO intubation, duration of ECPR, method of cannulation; baseline laboratory data at admission and the most aberrant laboratory test results during ECMO (>48 hours <7 days after ECMO).

The BALF samples collected from a subset of enrolled patients within 48 hours after ECMO assistance were tested with mNGS. Additionally, BALF samples collected either within 48 hours or between 48 hours and 7 days after ECMO assistance were subjected to routine culture. The specific results obtained from mNGS testing and BALF culture were recorded.

### 2.3 Definition and clinical outcomes

EOP was diagnosed when the patient met all clinical, radiological and microbiological criteria within 48h-7d after meeting ECPR (Ryu et al., 2015). Clinical criteria for EOP required the presence of at least two of the following: 1) fever ≥ 38.0°C (temperature in ECMO patients is typically regulated by extracorporeal circulation); 2) leukocytosis or leukopenia; and 3) purulent tracheobronchial secretions. Radiological criteria for EOP included new or progressive infiltrates or newly developed solid lesions on chest radiographs. Microbiological criteria involved the absence of normal positive respiratory cultures indicating a typical flora. Additionally, two independent physicians will validate the diagnosis of EOP to ensure accuracy and comparability. Prophylactic antibiotic use was defined as antibiotics given before signs of infection within 24 hours of ECPR. Time consumption for ECPR referred to the duration from preparation to establishment of adequate ECMO flow. The criteria for successful ECMO weaning were defined as achieving hemodynamic stability within 24 hours after withdrawal of ECMO and not requiring additional assistance from extracorporeal life support (ECLS).

The primary outcome of this study was the diagnosis of EOP, while the secondary outcomes included successful ECMO weaning and survival at discharge. Additionally, no cases of late-onset pneumonia were observed in non-EOP patients.

### 2.4 ECPR procedures

All patients received VA ECMO support (Maquet Cardiopulonic, Hirlingen, Germany) with percutaneous vascular access as the preferred method using the Seldinger technique due to its advantages of easy insertion and removal of cannulas. Surgical excisional exposure was considered a secondary option when percutaneous cannulation failed (Ryu et al., 2015; Vakil et al., 2021). The femoral vessel was used as the vascular access site in all cases with 19-25 French vein cannulas and 15-17 French artery cannulas. The cessation of cardiac compressions is recommended once the flow rate of VA ECMO reaches 3 L/min, indicating the establishment of sufficient circulation (Richardson et al., 2021). Unisolated heparin was used as an anticoagulant to maintain an activated clotting time (ACT) of 1.5 times the upper limit of normal and a partial thromboplastin time (APTT) of 40-55 seconds.

The ECMO flow rate are adjusted to meet the criteria of a patient with a cardiac index exceeding 2.2 L/min/m^2^, a central mixed venous oxygen saturation above 70%, and a mean arterial pressure higher than 65 mmHg (Park et al., 2014). Typically, an arterial catheter is utilized for continuous blood pressure monitoring. Once successfully initiated, prompt identification and treatment of the underlying cause of cardiac arrest are imperative. If a flow rate ranging from 0.5-1.0 L/min is achieved, consideration may be given to discontinuing VA ECMO support. However, each stage of flow reduction should be evaluated through echocardiography and hemodynamic assessment (Richardson et al., 2021).

### 2.5 mNGS methodology and process

After preprocessing of the BALF samples, microbial genomic DNA was extracted using the TIANamp Micro DNA kit (TIANGEN Biotech, Beijing) following the manufacturer’s instructions. Subsequently, DNA libraries were constructed through processes including DNA fragmentation, end-repair, 3’ adenylation, adapter ligation, PCR amplification and purification. The quality of the resulting libraries was assessed using an Agilent 2100 Bioanalyzer (Agilent, USA) and an ABI StepOnePlus real-time PCR system. Finally, qualified libraries were subjected to sequencing on a NextSeq 550Dx platform (Illumina, USA) with a read length of 75 bp.

Illumina Next 550 sequencers are utilized for macrogenomics sequencing, with each macrogenomics sequencing batch containing 15-20 samples and including one negative control. The internal reference is derived from Arabidopsis thaliana and is provided by the sequencing manufacturer. High-quality sequencing data were obtained by eliminating low-quality and short reads (<35bp in length), followed by the generation of strictly aligned reads to pathogen species (SDSMRN) and pathogen genus (SDSMRNG). The list of microorganisms acquired through the aforementioned analytical process was compared against an internal background database encompassing microorganisms present in over 50% of laboratory samples within the past three months. Suspected background microorganisms were excluded, while suspicious pathogens were identified as those exhibiting SDSMRN > 50 and at least three times higher than the control; however, suspicious pathogens with SDSMRN < 50 should have a minimum fivefold increase compared to the control.

### 2.6 Statistical analysis

Statistical analysis of all collected data was performed using SPSS for Windows (version 21.0; SPSS Inc., Chicago, IL, USA). Continuous variables were presented as mean ± standard deviation and compared using an unpaired t-test. Skewed continuous variables were expressed as quartiles and compared using the Mann-Whitney U test. Categorical variables were represented as frequency (composition ratio) and compared using Pearson’s χ test or Fisher’s exact test. Multicollinearity among predictors was assessed using variance inflation factors (VIFs). A VIF value exceeding 10 was considered indicative of significant collinearity. For the logistic regression analysis, we employed a binary logistic regression model to examine the relationship between the predictor variables and the outcome variable. *P*<0.05 indicates that the differences were statistically significant.

## 3 Results

### 3.1 Comparative analysis of patients undergoing ECPR based on ECMO weaning and survival status at discharge

The comparison of ECPR patients between the successful ECMO weaning group and the ECMO weaning failure group is presented in **Table 1**. ECPR patients who successfully weaned from ECMO exhibited lower SOFA scores on admission in comparison to those with failed weaning (11.89 ± 2.19 vs 13.58 ± 1.75, p<0.001, **Table 1**). Moreover, they had a shorter duration of ECPR consumption (31.78 ± 14.18 min vs 40.20 ± 18.61 min, p=0.031, **Table 1**), and had higher rates of mNGS testing (47.2% vs 15%, p=0.002, **Table 1**). Baseline lactate levels were lower in the success group compared to the failure group; however, this difference did not reach statistical significance level.

**Table 1:** The characteristics between the successful ECMO weaning group and the ECMO weaning failure group.

Variables	Total	Success	Failure	P Value
	(n=76)	(n=36)	(n=40)	
**Male**, (n%)	50(65.8)	21(58.3)	29(72.5)	0.194
**Age**, (y)	48.37 ± 16.02	50.33 ± 15.10	46.6 ± 16.80	0.314
**BMI**, (Kg/m2)	24.48 ± 3.03	24.17 ± 2.85	24.76 ± 3.19	0.404
**History of smoking**, (n%)	29 (38.2)	12 (33.3)	17(42.5)	0.411
Comorbidities, (n%)				
Hypertension	22 (28.9)	9 (25.0)	13 (32.5)	0.472
Type 2 diabetes	16 (21.1)	7 (19.4)	9 (22.5)	0.744
Dyslipidemia	3 (3.9)	1 (2.8)	2(5.0)	1.000
Primary disease, (n%)				
Acute myocardial infarction	47 (61.8)	21 (58.3)	26 (65.0)	0.096
Fulminant myocarditis	6 (7.9)	4 (11.1)	2 (5.0)	
Pulmonary embolism	9 (11.8)	7 (19.4)	2 (5.0)	
Others	14 (18.4)	4 (11.1)	10 (25.0)	
**IABP**, (n%)	25 (32.9)	15 (41.7)	10 (25.0)	0.123
SOFA Score*	12.78 ± 2.13	11.89 ± 2.19	13.58 ± 1.75	<0.001
ECPR related variables				
Time consuming of VA-ECMO cannulation, (min)	18.09 ± 9.01	17.19 ± 9.31	18.90 ± 8.77	0.414
Time consuming of ECPR*, (min)	36.21 ± 17.09	31.78 ± 14.18	40.20 ± 18.61	0.031
Shunt, (n%)	36 (47.4)	16 (44.4)	20 (50.0)	0.628
Methods of cannulation, (n%)				
Percutaneous puncture	64 (84.2)	32 (88.9)	32 (80.0)	0.289
Surgical incision	12 (15.8)	4 (11.1)	8 (20.0)	
Laboratory examinations				
White blood cell, (109/L)	14.37 ± 6.69	14.64 ± 5.58	14.13 ± 7.61	0.746
Red blood cell, (1012/L)	4.00 ± 0.98	3.92 ± 0.86	4.07 ± 1.08	0.509
Hemoglobin, (g/L)	121.48 ± 31.58	119.79 ± 29.02	123.00 ± 34.01	0.661
Platelet, (109/L)	206.38 ± 100.20	206.11 ± 79.14	206.63 ± 116.99	0.982
Alanine transaminase, (U/L)	51.50 (19.25-155.50)	67.50 (18.25-189.00)	38.50 (20.50-117.50)	0.529
Aspartate transaminase, (U/L)	69.50 (24.25-391.00)	132.95 (21.0-421.0)	60.50 (28.50-276.25)	0.831
Albumin, (g/L)	38.32 ± 26.69	39.75 ± 34.62	37.03 ± 17.05	0.660
Total bilirubin, (μmol/L)	10.65 (6.08-16.73)	9.05 (5.65-16.63)	11.10 (6.70-17.78)	0.308
Creatinine, (μmol/L)	109.66 ± 58.62	101.41 ± 54.74	117.08 ± 61.65	0.248
Prothrombin time, (s)	12.70 (11.20-17.33)	12.90 (11.08-15.35)	12.20 (11.20-18.03)	0.983
APTT, (s)	43.03 ± 25.46	45.62 ± 32.06	40.7 ± 17.67	0.403
NT-pro-BNP, (pg/ml)	714.80(186.00-7699.75)	497.99(120.20-5985.25)	1117.50(372.62-9022.00)	0.173
cTnI, (ng/ml)	0.46 (0.06-2.54)	0.46 (0.15-6.57)	0.47 (0.06-2.26)	0.696
CRP, (mg/L)	19.18 (3.66-59.28)	18.91 (3.54-51.16)	20.31 (4.83-73.52)	0.913
Procalcitonin, (ng/ml)	0.30 (0.08-4.12)	0.26 (0.08-3.68)	0.31 (0.07-5.49)	0.603
Lactate, (mmol/L)	10.24 ± 5.75	9.08 ± 5.15	11.29 ± 6.11	0.095
**EOP**, (n%)	27 (35.5)	11 (30.6)	16 (40.0)	0.390
mNGS*, (n%)	23 (30.3)	17 (47.2)	6 (15.0)	0.002
Survival*, (n%)	24 (31.6)	24 (66.7)	0	<0.001

The data was shown as the mean ± SD, median (interquartile 25-75) or n (percentage). ^*^ Indicate significant difference (^*^ indicate p<0.05). IHCA, in-hospital cardiac arrest; ECPR, extracorporeal cardiopulmonary resuscitation; BMI, body mass index; IABP, intra-aortic balloon pump; SOFA, sequential organ failure assessment; ECMO, extracorporeal membrane oxygenation; APTT, activated partial thromboplastin time; CRP, C-reactive protein; EOP, early onset pneumonia; mNGS, metagenomic next-generation sequencing.

The comparison of ECPR patients based on survival status at discharge is presented in **Table 2**. The survival group exhibited a lower incidence of CRRT (34.8% vs 66.0%, p=0.014, **Table 2**), higher utilization of mNGS testing (54.2% vs 19.2%, p=0.002, **Table 2**), greater success in ECMO weaning (100% vs 23.1%, p<0.001, **Table 2**), shorter duration of ECPR consumption (29.75 ± 13.15 min vs 39.19 ± 17.96 min, p=0.024, **Table 2**), and lower SOFA scores (10.96 ± 1.88 vs 13.62 ± 1.68, p<0.001, **Table 2**) compared to the non-survival group at discharge. Laboratory examinations upon admission revealed lower levels of creatinine (89.66 ± 43.75 μmol/L vs 118.89 ± 62.58 μmol/L, p=0.043, **Table 2**) and lactate (7.35 ± 4.82 mmol/L vs 11.57 ± 5.69 mmol/L, p=0.002, **Table 2**) in the survival group compared to the non-survival group. During the period >48h and within 7d after ECMO, the most aberrant laboratory examinations showed lower WBC counts [(9.49 ± 3.80)×10^9^/L vs (12.80 ± 6.57)×10^9^/L, p=0.024, **Table 2**], ALT [85.50 (52.50-164.75)U/L vs 238.50 (90.00-754.00)U/L, p=0.003, **Table 2**], AST[142.50 (83.50-221.00)U/L vs 447.50 (119.75-1683.25)U/L, p=0.001, **Table 2**], creatinine (133.51 ± 89.93 μmol/L vs 187.61 ± 89.24 μmol/L, p=0.017, **Table 2**), and NT-pro-BNP [2785 (738.5-5620.05)pg/ml vs 5566.00 (1720.75-11828.50)pg/ml, p=0.024, **Table 2**] levels in the survival group compared to the non-survival group.

**Table 2:** The characteristics between the survivors and the non-survivors at discharge.

Variables	Survivors	Non-survivors	P Value
	(n=24)	(n=52)	
**Male**, (n%)	14 (58.3)	36 (69.2)	0.352
**Age**, (y)	51.13 ± 13.99	47.1 ± 16.86	0.311
**BMI**, (Kg/m^2^)	24.07 ± 2.46	24.67 ± 3.27	0.428
**History of smoking**, (n%)	7 (29.2)	22 (42.3)	0.273
Comorbidities, (n%)			
Hypertension	6 (25.0)	16 (30.8)	0.606
Type 2 diabetes	4 (16.7)	12 (23.1)	0.524
Dyslipidemia	1 (4.2)	2 (3.8)	1.000
Primary disease, (n%)			
Acute myocardial infarction	15 (62.5)	32 (61.5)	0.312
Fulminant myocarditis	3 (12.5)	3 (5.8)	
Pulmonary embolism	4 (16.7)	5 (9.6)	
Others	2 (8.3)	12 (23.1)	
**IABP**, (n%)	10 (41.7)	15 (28.8)	0.269
SOFA Score***	10.96 ± 1.88	13.62 ± 1.68	<0.001
ECPR related variables			
Time consuming of VA-ECMO cannulation, (min)	18.04 ± 9.67	18.12 ± 8.79	0.974
Time consuming of ECPR*, (min)	29.75 ± 13.15	39.19 ± 17.96	0.024
Shunt, (n%)	11 (45.8)	25 (48.1)	0.856
Methods of cannulation, (n%)			
Percutaneous puncture	21 (87.5)	43 (82.7)	0.845
Surgical incision	3 (12.5)	9 (17.3)	
Laboratory examinations			
White blood cell, (10^9^/L)	13.59 ± 5.89	14.73 ± 7.05	0.494
Red blood cell, (10^12^/L)	3.89 ± 0.95	4.06 ± 1.00	0.491
Hemoglobin, (g/L)	119.00 ± 30.20	122.63 ± 32.42	0.645
Platelet, (10^9^/L)	210.08 ± 66.42	204.67 ± 112.99	0.828
Alanine transaminase, (U/L)	52.50 (18.25-172.00)	51.50 (20.50-150.50)	0.767
Aspartate transaminase, (U/L)	60.00 (18.50-421.00)	77.00 (28.50-308.75)	0.374
Albumin, (g/L)	42.79 ± 42.00	36.25 ± 15.43	0.324
Total bilirubin, (μmol/L)	8.20 (5.65-15.58)	11.10 (6.48-17.85)	0.232
Creatinine*, (μmol/L)	89.66 ± 43.75	118.89 ± 62.58	0.043
Prothrombin time, (s)	12.70 (10.73-14.50)	12.70 (11.25-18.20)	0.368
APTT, (s)	48.85 ± 37.75	40.34 ± 16.95	0.301
NT-pro-BNP, (pg/ml)	460.40 (112.25-3847.72)	1117.50 (304.28-9233.25)	0.089
cTnI, (ng/ml)	0.46 (0.15-5.76)	0.47 (0.06-2.54)	0.737
CRP, (mg/L)	25.18 (3.44-69.99)	18.34 (3.76-59.28)	0.542
Procalcitonin, (ng/ml)	0.36 (0.12-5.22)	0.25 (0.07-3.82)	0.337
Lactate**, (mmol/L)	7.35 ± 4.82	11.57 ± 5.69	0.002
Most abnormal value of laboratory examinations during ECMO (>48 hours & <7 days after ECMO)			
White blood cell*, (10^9^/L)	9.49 ± 3.80	12.80 ± 6.57	0.024
Red blood cell, (10^12^/L)	3.19 ± 0.50	2.95 ± 0.62	0.099
Hemoglobin, (g/L)	97.68 ± 15.57	89.69 ± 20.17	0.090
Platelet, (10^9^/L)	92.75 ± 38.07	83.37 ± 51.06	0.425
Alanine transaminase**, (U/L)	85.50 (52.50-164.75)	238.50 (90.00-754.00)	0.003
Aspartate transaminase**, (U/L)	142.50 (83.50-221.00)	447.50 (119.75-1683.25)	0.001
Creatinine*, (μmol/L)	133.51 ± 89.93	187.61 ± 89.24	0.017
NT-pro-BNP*, (pg/ml)	2785 (738.5-5620.05)	5566 (1720.75-11828.50)	0.024
cTnI, (ng/ml)	4.75 (0.48-8.45)	2.61 (0.56-9.95)	0.783
CRP, (mg/L)	69.30 (18.68-105.56)	70.97 (17.68-160.93)	0.631
Procalcitonin, (ng/ml)	3.45 (0.47-14.16)	7.01 (1.50-21.45)	0.151
**EOP**, (n%)	5 (20.8)	22 (42.3)	0.069
mNGS**, (n%)	13 (54.2)	10 (19.2)	0.002
ECMO weaning success***, (n%)	24 (100)	12 (23.1)	<0.001

The data was shown as the mean ± SD, median (interquartile 25-75) or n (percentage). ^*^ Indicate significant difference (^*^ indicate p<0.05, ^**^ indicate p<0.01, ^***^ indicate p<0.001). BMI, body mass index; IABP, intra-aortic balloon pump; SOFA, sequential organ failure assessment; VA-ECMO, veno-arterial extracorporeal membrane oxygenation; ECPR, extracorporeal cardiopulmonary resuscitation; APTT, activated partial thromboplastin time; CRP, C-reactive protein; EOP, early onset pneumonia; mNGS, metagenomic next-generation sequencing.

### 3.2 Characteristics of EOP and non-EOP patients

Compared to non-EOP patients, EOP patients exhibited significantly higher SOFA scores (13.70 ± 1.86 vs 12.27 ± 2.12, p=0.004, **Table 3**), longer duration of ECPR consumption (42.41 ± 17.20 min vs 32.80 ± 16.20 min, p=0.021, **Table 3**), lower rate of mNGS testing (11.1% vs 40.8%, p=0.007, **Table 3**), and elevated levels of CRP, PCT and WBC counts (the highest CRP, PCT and WBC counts during the period >48h and within 7d after ECMO) [CRP: 99.80 (58.20-171.50) mg/dl vs 34.19 (9.77-103.28) mg/dl, p =0.003; PCT: 12.52 (4.36-25.78) ng/ml vs 3.57 (0.38-15.77) ng/ml, p =0.003; WBC counts: (16.62 ± 6.19) ×10^9^/L vs (9.08 ± 3.89)×10^9^/L, p<0.001; respectively, **Table 3**] compared to non-EOP individuals.

**Table 3:** Characteristics of ECPR patients with or without EOP.

Variables	EOP	Non-EOP	P Value
	(n=27)	(n=49)	
**Male**, (n%)	17 (63.0)	33 (67.3)	0.700
**Age**, (y)	49.26 ± 16.31	47.88 ± 16.01	0.722
SOFA Score**	13.70 ± 1.86	12.27 ± 2.12	0.004
Time consuming of ECPR*, (min)	42.41 ± 17.20	32.80 ± 16.20	0.018
Laboratory examinations at ECMO onset			
White blood cell, (10^9^/L)	15.82 ± 7.97	13.57 ± 5.79	0.161
Red blood cell, (10^12^/L)	4.24 ± 1.07	3.87 ± 0.92	0.125
Hemoglobin, (g/L)	127.74 ± 34.13	118.03 ± 29.88	0.202
Platelet, (10^9^/L)	199.07 ± 111.31	210.41 ± 94.50	0.640
Alaninetransaminase, (U/L)	40.00 (22.00-214.00)	61.00 (18.50-128.00)	0.799
Aspartate transaminase, (U/L)	74.00 (27.00-358.00)	68.00 (22.50-405.50)	0.741
Creatinine, (μmol/L)	115.82 ± 50.28	106.26 ± 62.99	0.500
NT-pro-BNP, (pg/ml)	1639.00 (176.00-8820.00)	574.00 (187.00-7293.50)	0.629
cTnI, (ng/ml)	0.43 (0.06-2.00)	0.50 (0.06-3.92)	0.543
CRP, (mg/L)	19.41 (3.51-48.08)	18.95 (3.80-76.23)	0.753
Procalcitonin, (ng/ml)	0.26 (0.06-5.56)	0.31 (0.08-3.81)	0.905
Lactate, (mmol/L)	10.66 ± 5.39	10.01 ± 5.98	0.638
Most abnormal value of laboratory examinations during ECMO (>48 hours & <7 days after ECMO)			
White blood cell***, (10^9^/L)	16.62 ± 6.19	9.08 ± 3.89	<0.001
Red blood cell, (10^12^/L)	3.02 ± 0.53	3.03 ± 0.63	0.931
Hemoglobin, (g/L)	91.28 ± 16.96	92.73 ± 20.34	0.754
Platelet, (10^9^/L)	92.07 ± 59.98	83.16 ± 38.94	0.491
CRP**, (mg/L)	99.80 (58.20-171.50)	34.19 (9.77-103.28)	0.003
Procalcitonin**, (ng/ml)	12.52 (4.36-25.78)	3.57 (0.38-15.77)	0.003
**ECMO weaning success, (n%)**	11 (40.7)	25 (51.0)	0.390
mNGS*, (n%)	3 (11.1)	20 (40.8)	0.007
**Prophylactic antibiotics use**, (n%)	26 (96.3)	41 (83.7)	0.103
**Survivor**, (n%)	5 (18.5)	19 (38.8)	0.069

The data was shown as the mean ± SD, median (interquartile 25-75) or n (percentage). ^*^ Indicate significant difference (^*^ indicate p<0.05, ^**^ indicate p<0.01, ^***^ indicate p<0.001). ECPR, extracorporeal cardiopulmonary resuscitation; EOP, early onset pneumonia; SOFA, sequential organ failure assessment; ECMO, extracorporeal membrane oxygenation; CRP, C-reactive protein; mNGS, metagenomic next-generation sequencing.

The gender, BMI, comorbidities, baseline levels of white blood cell counts, red blood cell counts, hemoglobin, platelet counts, alanine transaminase, aspartate transaminase, creatinine, NT-proBNP, cTnI, CRP, procalcitonin and lactate; the most abnormal levels of red blood cell counts, hemoglobin and platelet counts during the period >48h and within 7d after ECMO assistance; the rate of successful weaning from ECMO; and percentages of prophylactic antibiotic use exhibited no statistically significant differences between EOP patients and non-EOP patients (all p>0.05; **Table 3**).

### 3.3 Details of mNGS assay

**Figure 2:**
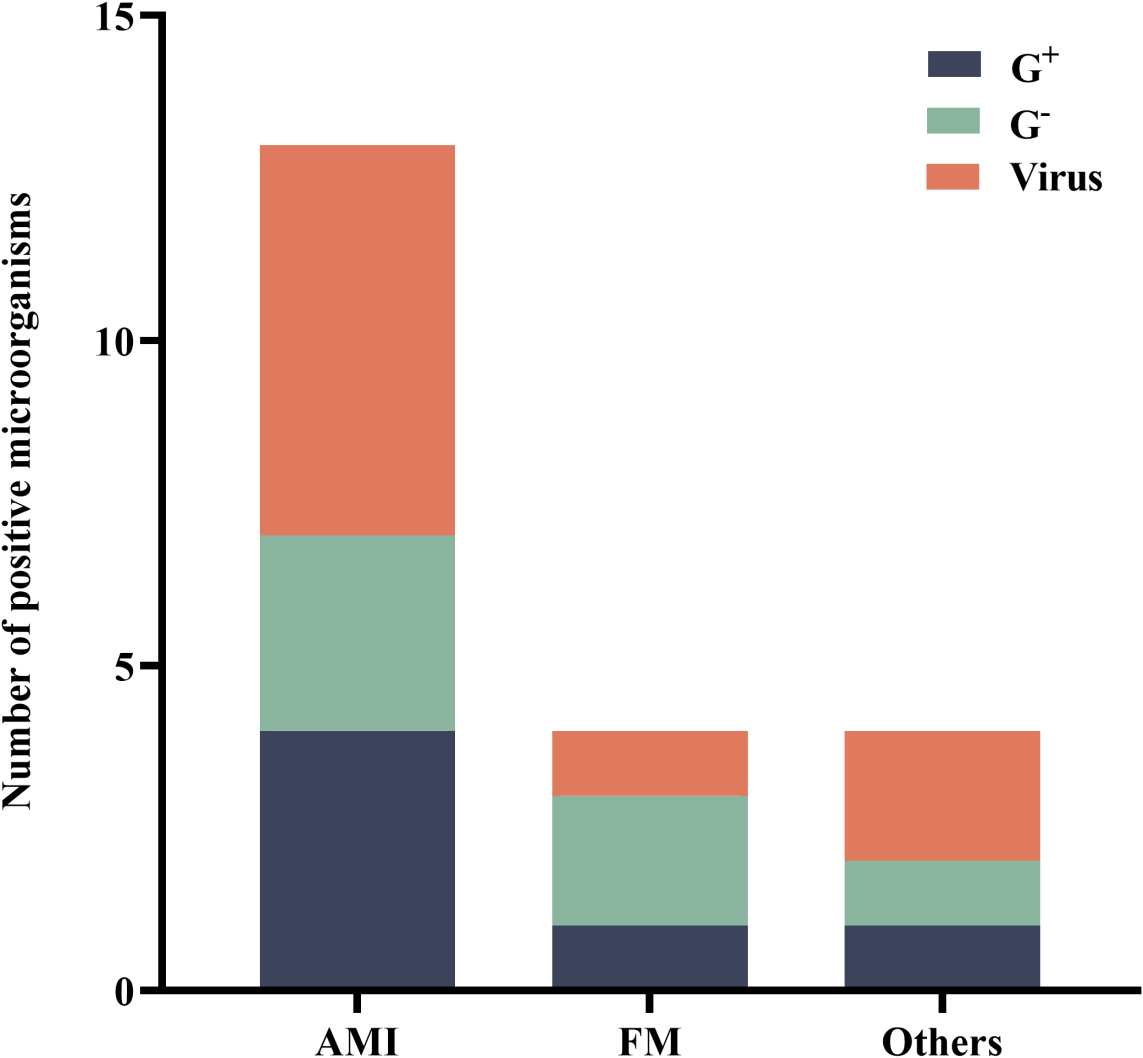
Distribution of microorganisms that tested positive for mNGS. AMI, acute myocardial infarction; FM, fulminant myocarditis; G^+^, Gram-positive bacteria; G^-^, Gram-negative bacteria; mNGS, metagenomic next-generation sequencing.

Among the 76 patients who underwent ECPR, BALF was tested using mNGS within 48 hours in a subset of 23 individuals (30.3%). Within this subgroup, acute myocardial infarction (AMI) was identified as the primary cause in 16 patients (69.6%), while fulminant myocarditis accounted for the primary cause in 3 patients (13.0%). Furthermore, four patients (17.4%) were diagnosed with other conditions such as poisoning and smog exposure. Among these cases, eleven patients (47.8%) yielded positive mNGS results. Subsequently, we performed a specific analysis to identify the presence of pathogens associated with each respective primary disease (**Figure 2**).

The pathogens detected by mNGS (within 48 hours during ECMO) included bacteria and viruses, predominantly consisting of oropharyngeal colonizing bacteria. Specifically, the detected strains comprised 4 strains of *Haemophilus parainfluenzae*, 2 strains of *Streptococcus pneumoniae*, 1 strain each of S*treptococcus anginosus* and *Streptococcus mitis*, as well as single strains of *Staphylococcus hominis*, *Gemella morbillorum*, *Haemophilus influenzae*, *Dialister pneumosintes*. Additionally, there were 3 strains of *Human alpha herpes virus 1*, 2 strains of *Human beta herpes virus 5*, along with single strains of *Epstein-Barr virus*, *Human parainfluenza virus 3*, *Cyto Megalo Virus* and *Torque teno virus type29*.

The positive pathogens detected were classified based on the primary disease. Among 16 patients with AMI, a total of 4 (30.8%) Gram-positive strains, 3 (23.1%) Gram-negative strains, and 6 (46.2%) viral strains were identified. In the case of fulminant myocarditis in 3 patients, one (25.0%) Gram-positive strain, two (50.0%) Gram-negative strains, and one (25.0%) viral strain were detected.

### 3.4 Comparison of mNGS assay and BALF culture results

**Figure 3:**
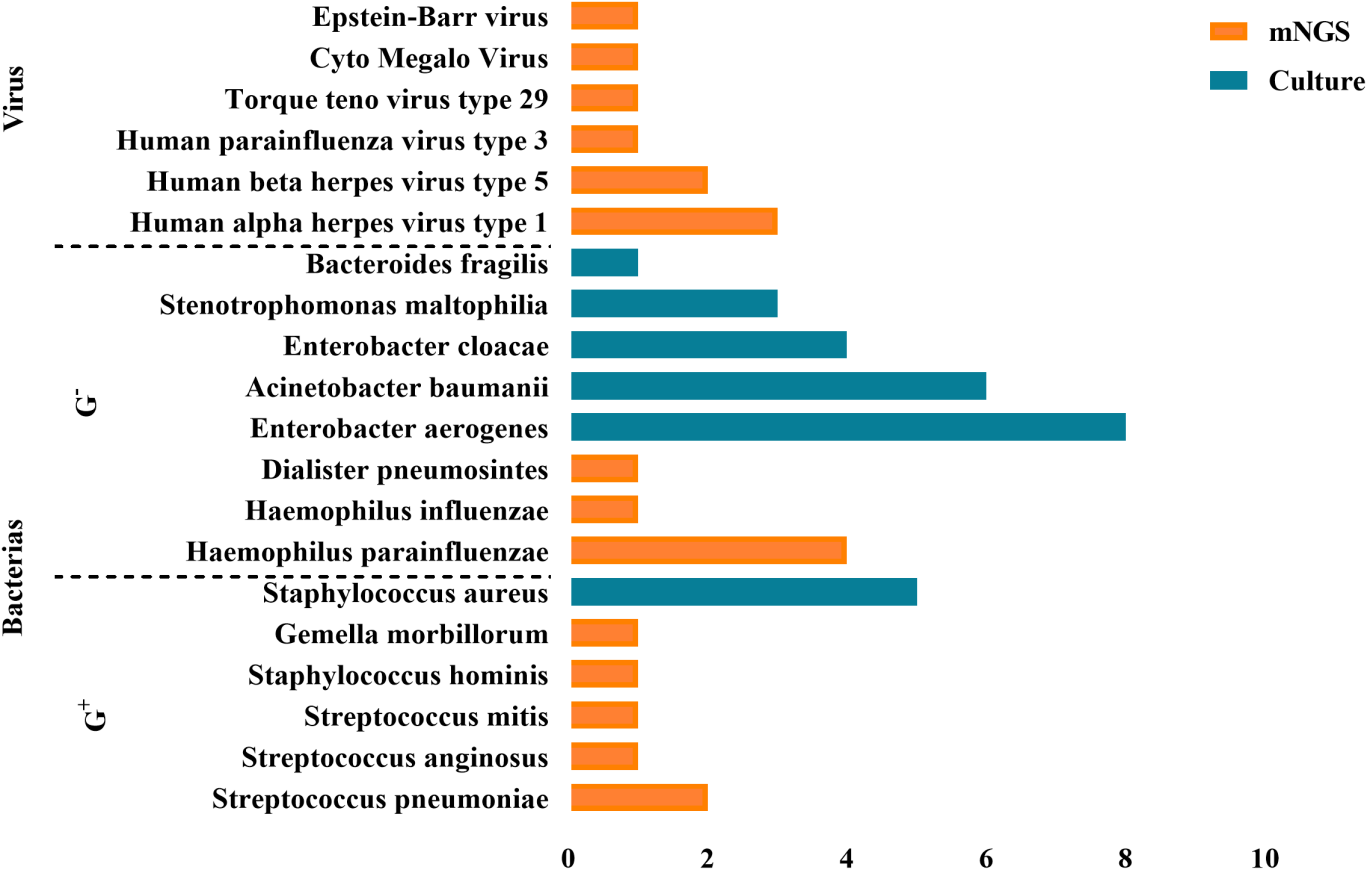
Positive pathogens detected by mNGS and BALF culture. A total of 48 pathogens were detected, including 39 bacteria, 9 viruses.

BALF culture results performed in parallel (<48h) with the mNGS test revealed no bacterial or other pathogenic growth. However, 48 hours to 7 days after ECMO onset, all EOP patients’ BALF cultures were positive. As shown in **Figure 3**, there were eight strains of *Enterobacter aerogenes*, six strains of Acinetobacter baumanii, four strains of *Enterobacter cloacae*, three strains of *Stenotrophomonas maltophilia*, one strain of *Bacteroides fragilis*, and five strains of *Staphylococcus aureus*.

The comparison between mNGS and BALF culture results revealed that mNGS detected a higher proportion of Gram-positive bacteria and viruses as pathogens, whereas BALF culture results showed a higher proportion of Gram-negative bacteria.

### 3.5 Predictors of clinical outcomes

Logistic regression was employed to analyze the predictors of ECMO weaning failure, mortality at discharge, and the incidence of EOP.

Ultimately, patients with lower SOFA scores on admission [OR (95%CI): 1.447 (1.107-1.890), p=0.007, **Table 4**] and those who underwent early mNGS testing within 48 hours after ECPR [OR (95%CI): 0.273 (0.086-0.865), p=0.027, **Table 4**] demonstrated a higher probability of successful weaning from ECMO.

**Table 4:** Univariate and multivariate logistic regression analysis of predictors of ECMO weaning failure, mortality at discharge, and the incidence of EOP.

Variables	Univariate regression analysis		Multivariate regression analysis	
	OR (95%CI)	P Value	OR (95%CI)	P Value
ECMO weaning failure patients with ECPR				
**SOFA Score**	1.540 (1.185-2.002)	0.001	1.447 (1.107-1.890)	0.007
**mNGS**	0.197 (0.067-0.585)	0.003	0.273 (0.086-0.865)	0.027
**ECPR time**	1.033 (1.002-1.066)	0.039		
**Lactate levels**	1.072 (0.987-1.165)	0.097		
Mortality at discharge				
**SOFA Score**	2.409 (1.569-3.700)	<0.001	2.404 (1.422-4.064)	0.001
**ECPR time**	1.043 (1.004-1.084)	0.032	1.051 (0.985-1.122)	0.133
**mNGS**	0.201 (0.07-0.581)	0.003	0.209 (0.042-1.041)	0.056
**Lactate levels**	1.161 (1.048-1.287)	0.004	1.176 (1.017-1.361)	0.029
**NT-pro-BNP**	1.000 (1.000-1.000)	0.034	1.000 (1.000-1.000)	0.177
**Creatinine**	1.007 (1.001-1.014)	0.022		
**White blood cell**	1.141 (1.012-1.286)	0.031		
**Alanine transaminase**	1.002 (1.000-1.003)	0.055		
Incidence of EOP				
**mNGS**	0.181 (0.048-0.684)	0.012	0.186 (0.035-0.979)	0.047
**ECPR time**	1.035 (1.004-1.066)	0.026	1.035 (0.999-1.073)	0.053
**SOFA Score**	1.432 (1.104-1.856)	0.007	1.341 (0.999-1.800)	0.051
**CRP levels**	1.008 (1.002-1.013)	0.010	1.011 (1.003-1.019)	0.006
**PCT levels**	1.014 (0.996-1.033)	0.131		

The levels of NT-pro-BNP, creatinine, white blood cells, alanine aminotransferase, CRP, and PCT were determined as the most aberrant values observed during ECMO (>48 hours & <7 days after ECMO). mNGS, metagenomic next-generation sequencing; IHCA, in-hospital cardiac arrest; ECPR, extracorporeal cardiopulmonary resuscitation; EOP, early onset pneumonia; SOFA, sequential organ failure assessment; CRP, C-reactive protein; PCT, Procalcitonin; WBC, Procalcitonin; ECMO, extracorporeal membrane oxygenation; OR, odds ratio; CI, confidence interval.

Patients with higher SOFA scores on admission [OR (95%CI): 2.404 (1.422-4.064), p=0.001, **Table 4**], and elevated lactate levels [OR (95%CI): 1.176 (1.017-1.361), p=0.029, **Table 4**] exhibited an increased likelihood of mortality at discharge.

Furthermore, early mNGS detection [OR (95%CI): 0.186 (0.035-0.979), p=0.047, **Table 4**], and lower CRP levels (48h-7d after ECMO) [OR (95%CI):1.011 (1.003-1.019), p=0.006, **Table 4**] were associated with a reduced incidence of EOP.

## 4 Discussion

In the present study investigating patients who underwent ECPR following IHCA, early mNGS testing of BALF samples was associated with a reduced occurrence of EOP and improved successful weaning from ECMO.

In the logistic regression model, early mNGS testing was found to be an independent negative predictor of EOP development and an independent positive predictor of successful withdrawal of ECMO in patients undergoing ECPR. Nosocomial infections significantly contribute to mortality among critically ill patients admitted to hospitals, particularly those who have experienced cardiac arrest. The reported incidence of hospital-acquired infections in ECMO patients varies widely in the literature, ranging from 20% to 60% (Schmidt et al., 2012; Grasselli et al., 2017; Austin et al., 2017; Juthani et al., 2018; Bizzarro et al., 2011). ECPR patients often undergo various invasive procedures such as tracheal intubation, placement of indwelling ECMO cannula, deep venous puncture, CRRT and IABP, which substantially increase the risk of infection (Vincent et al., 1995). In our study, BALF samples were collected within 48 hours after initiation of ECMO from IHCA patients and subjected to routine culture and mNGS testing. The results revealed that routine cultures yielded negative findings for BALF samples; however, mNGS detected multiple pathogens including bacteria and viruses with a predominance of viral species and oral colonizing bacteria. Between 48 hours and 7 days after ECMO initiation, BALF cultures from ECPR patients demonstrated the presence of multiple pathogenic bacteria including multi-drug resistant (MDR) strains such as *Acinetobacter baumannii* and *methicillin-resistant Staphylococcus aureus* (MRSA).

The infection caused by MDR pathogens has been demonstrated to be a significant risk factor for increased mortality in ECPR patients admitted to the intensive care unit (ICU), and is also associated with higher costs of ICU admission (Neidell et al., 2012; Depuydt et al., 2008). Due to the prolonged use of antibiotics, patients in the ICU are particularly susceptible to MDR pathogen infections. In our study, most of the ECPR patients received prophylactic antibiotic therapy but inevitably developed MDR pathogenic infections. Therefore, it is crucial for us to address how we can effectively prevent and reduce MDR infections. In this study, we have introduced a novel diagnostic test that facilitates the identification of pathogen distribution among patients undergoing ECPR following IHCA, and evaluates its diagnostic efficacy for EOP.

The mNGS test has been widely employed in clinical practice. In the context of infectious diseases, the mNGS test exhibits exceptional sensitivity, a short testing cycle, and broad applicability (Brown et al., 2018; Carpenter et al., 2019). Moreover, it obviates the need for pathogenic bacterial isolation and remains unaffected by antibiotic usage, thereby reducing false negative rates. Importantly, mNGS analysis holds the potential to detect all nucleotide sequences within a sample theoretically enabling simultaneous analysis of multiple pathogens. A recent investigation demonstrated that mNGS of BALF samples could yield more precise diagnostic information for lung infections (Chen et al., 2021). In our study cohort, patients who underwent early mNGS testing exhibited significantly higher success rates in ECMO withdrawal and notably lower incidence of EOP. Additionally, the small proportion of Gram-positive bacteria shown by the mNGS test may be due to the fact that the majority of prophylactic antibiotic applications were broad-spectrum antibiotics targeting Gram-positive bacteria.

The incidence of MDR can be reduced by shortening the duration of prophylactic antibiotic administration for individuals without detectable causative organisms. Conversely, individuals with identified suspected causative organisms should adjust their dosing regimen to precisely treat existing infections and prevent the emergence of medically-induced MDR infections resulting from antibiotic misuse. Additionally, patients undergoing ECPR exhibit heightened levels of inflammatory factors and may even develop a systemic inflammatory response (Stub et al., 2011; Wengenmayer et al., 2017; Millar et al., 2016). Inflammation plays a pivotal role in the pathogenesis of EOP. Not only does infection directly contribute to EOP, but it also exacerbates inflammation, thereby promoting the development of EOP and potentially influencing the success of ECMO withdrawal. This study reaffirmed the association between inflammation and EOP. Multivariate logistic regression analysis demonstrated that CRP serves as one of the predictors for EOP development, consistent with previous research findings (Shiba et al., 2020). Although PCT levels were significantly higher in the EOP group compared to the non-EOP group, they did not independently predict EOP occurrence, aligning with prior studies (Mongardon et al., 2010; Zielińska-Borkowska et al., 2012).

Furthermore, our study confirms previous findings regarding the relationship between SOFA score and mortality rate at charge, ECPR time-consuming and incidence of EOP, which is consistent with previous studies (Haas et al., 2017; Rietdijk et al., 2020). The lack of significant correlation between ECPR time-consuming and mortality may be attributed to limited sample size considerations. In recent years, lactate has emerged as a critical predictor for assessing the risk of death and neurological outcomes in patients undergoing cardiac arrest (Bartos et al., 2020; Hayashida et al., 2017). Pre-ECPR lactate levels have been increasingly recognized as a potential prognostic marker for mortality, supported by several studies (Bertic et al., 2022; Dennis et al., 2017; Halenarova et al., 2022). However, post-ECPR, the use of lactate clearance as a prognostic marker remains debated. A cohort study on ECPR patients reported limited prognostic value for lactate clearance in predicting mortality (Jung et al., 2019). Conversely, an international multicenter study highlighted the association between pre-ECPR lactate levels, lactate clearance within 24 hours of ECPR initiation, and one-year survival in refractory cardiac arrest patients. They found a significant, dose-dependent correlation between lactate clearance and one-year survival (Thevathasan et al., 2024). Our study identified peak lactate levels post-ECPR as an independent predictor of mortality at discharge. However, we acknowledge that our study did not specifically analyze lactate clearance post-ECPR, which limits our comprehensive understanding of lactate dynamics in post-ECPR prognosis.

The current study has several limitations. Firstly, the retrospective design and small sample size present significant limitations that affect the generalizability of our findings. The retrospective nature of the study means that the data was collected from existing records, which may introduce inconsistencies and incomplete information not originally intended for the current research focus. This could lead to inaccuracies and limit the applicability of our results to other settings or populations. Secondly, considering the financial burden associated with mNGS testing, a majority of the patients tested were critically ill. Finally, only 23 patients underwent mNGS within 48 hours after ECPR, out of which only 11 BALF specimens yielded pathogen detection. Therefore, it is crucial to emphasize the significance of mNGS testing following ECPR and advocate for multiple repetitions of mNGS testing along with comparison to conventional cultures.

## 5 Conclusion

In patients treated with ECPR following IHCA, early mNGS testing to clarify microbial flora facilitates timely adjustments of antibiotic regimens, potentially reducing the incidence of EOP and improving short-term patient prognosis.

## Data Availability

The datasets presented in this study can be found in online repositories. The names of the repository/repositories and accession number(s) can be found below: https://www.ebi.ac.uk/ena, PRJEB72354.
